# BMC Complementary and alternative medicine reviewer acknowledgements, 2014

**DOI:** 10.1186/s12906-015-0518-6

**Published:** 2015-03-28

**Authors:** Thomas A Rowles

**Affiliations:** BioMed Central, Floor 6, 236 Gray’s Inn Road, London, WC1X 8HB UK

**Omar Abdel Salam**

Egypt

**Roslida Abdul Hamid**

Malaysia

**Aisha Abou Zeid**

Egypt

**Kamal-Eldin Abou-Elhamd**

Egypt

**Sawsan Abuhamdah**

Jordan

**Abuduaini Abulimiti**

USA

**Sandra Adams**

USA

**Salmon Adebayo**

South Africa

**Adewalw Adetutu**

Nigeria

**Oluyomi Adeyemi**

Nigeria

**Hadi Adibi**

Iran

**Neda Adibpour**

Iran

**Bulus Adzu**

Nigeria

**Abhinav Aeron**

India

**Eman Afify**

Egypt

**Muhammad Afzal**

India

**Gabriel Agbor**

Cameroon

**Bharat Aggarwal**

USA

**Bharat Aggarwal**

USA

**Riaz Ahmad**

India

**Sarfaraz Ahmad**

USA

**Amirhossein Ahmadi**

Iran

**Akefeh Ahmadiafshar**

Iran

**Augustine Ahmadu**

Nigeria

**Danish Ahmed**

India

**Khaled Ahmed**

Malaysia

**Mohamed Ahmed**

Egypt

**Osama Ahmed**

Egypt

**Kyung-Seop Ahn**

South Korea

**Mikel Aickin**

USA

**Nuriye Akev**

Turkey

**Dewan Akhter**

Bangladesh

**Hassan Akrami**

Iran

**Ali Al-Ahmad**

Germany

**Amin Alasbahi**

Yemen

**Angel Alex**

India

**Matthew Alexander**

USA

**Mohammed Al-Hariri**

Saudi Arabia

**Mahmoud Alhosin**

France

**Ehab Ali**

Egypt

**Nahid Ali**

India

**Niaz Ali**

Pakistan

**Md Ramjan Ali**

Bangladesh

**Mohammed Ali-Shtayeh**

Palestinian Territory

**Eman Alissa**

Saudi Arabia

**Aishah Al-Jarallah**

Kuwait

**Angel Alonso**

Mexico

**Terje Alraek**

Norway

**Celuta Alviano**

Brazil

**Adel Al-Zubairi**

Saudi Arabia

**Amro Amara**

Egypt

**Angela Amedee**

USA

**Mehrnoush Amid**

Malaysia

**Oluwatoyin Amira**

Nigeria

**Madhu Anand-Srivastava**

Canada

**Shyam Sunder Anchuri**

India

**Joel Anderson**

USA

**Ana Andreazzi**

Brazil

**Ofélia Anjos**

Portugal

**Shahid Ansari**

India

**Emmanouil Apostolidis**

USA

**Chippada Apparao**

India

**Danielle Aragão**

Brazil

**Sandra Arandelovic**

Serbia

**Jesil Aranjani**

India

**Adeyemi Oladapo Aremu**

South Africa

**Jann Arends**

Germany

**Jann Arends**

Germany

**Ebru Arioglu Inan**

Turkey

**Nanfack Donfack Arno Rusel**

Cameroon

**Ahmed Aroke Shahid**

Nigeria

**Ami Aronheim**

Israel

**Protus Arrey Tarkang**

Cameroon

**Myriam Arriaga-Alba**

Mexico

**Majid Artus**

United Kingdom

**Kantha Deivi Arunachalam**

India

**Mohammed Asad**

Saudi Arabia

**Yoshihisa Asada**

Japan

**Atsushi Asakura**

USA

**George Asare**

Ghana

**Gary Asher**

USA

**Sadegh Ashkani**

Malaysia

**Mohamed Ashour**

Egypt

**Sinem Aslan Erdem**

Turkey

**Özlem Sultan Aslantürk**

Turkey

**Stelios Assimakopoulos**

Greece

**Gilbert Ateufack**

Cameroon

**Ehud Atoun**

Israel

**Jithan Aukunuru**

India

**Mohammed Auwal Ibrahim**

Nigeria

**Suresh Awale**

Japan

**Maurice D Awouafack**

Cameroon

**Muhammad Ayaz**

Pakistan

**Boshra Azadi**

Iran

**Ulken Tunga Babaoglu**

Turkey

**Sitesh C Bachar**

Bangladesh

**Soo Kyung Bae**

South Korea

**Yoe-Sik Bae**

South Korea

**Margarete Dulce Bagatini**

Brazil

**Victor Bagla**

South Africa

**Puneet Kumar Bagri**

India

**Jie Bai**

China

**Yanhong Bai**

China

**Inkyung Baik**

South Korea

**Vivek K. Bajpai**

South Korea

**Usman Bala**

Malaysia

**Prabha Balaram**

Malaysia

**Ewa Balcerczak**

Poland

**Matthew Bambling**

Australia

**Heejung Bang**

USA

**Parveen Bansal**

India

**Yogendra Kumar Bansal**

India

**Jennifer Barber-Singh**

USA

**Sanaa Bardaweel**

Jordan

**Jo Barnes**

New Zealand

**Matthew Philip Greig Barnett**

New Zealand

**Luis Isamu Barros Kanzaki**

Brazil

**Silpi Basak**

India

**Ronan Batista**

Brazil

**Maurizio Battino**

Italy

**Saw Bawm**

Myanmar

**P S Bedi**

India

**Tania Begum**

Sweden

**Florian Beissner**

Germany

**Daniela Benedec**

Romania

**Kirsten Benkendorff**

Australia

**Alan Bensoussan**

Australia

**Iris Benzie**

Hong Kong

**Vance Berger**

USA

**Liron Berkovich**

Israel

**Dominique Bernard-Gallon**

France

**Luciano Bernardi**

Italy

**H Tanju Besler**

Turkey

**Thoudam Bhaigyabati**

India

**Supriya Bhalerao**

India

**Sowmya Bharani**

United Kingdom

**Khursheed Bhat**

India

**Piyali Bhattacharyya**

Puerto Rico

**Tapan Bhattacharyya**

Afghanistan

**Zhaoxiang Bian**

Hong Kong

**Stephen Birch**

Netherlands

**Tony Bird**

Australia

**Tannaz Birdi**

India

**Diane Birt**

USA

**Anupam Bishayee**

USA

**Felicity Bishop**

United Kingdom

**Anne Blais**

France

**Liesl Blott**

Australia

**Liu Bo**

China

**Ebru Bodur**

Turkey

**Friedrich Boege**

Germany

**Katja Boehm**

Germany

**Pompei Bolfa**

Saint Kitts and Nevis

**Maria Bonfleur**

Brazil

**Thidarut Boonmars**

Thailand

**Sandra Bosco**

Brazil

**R Beklem Bostancioglu**

Turkey

**Eliete Bouskela**

Brazil

**Fabrice Boyom**

Cameroon

**Fabrice Boyom**

Cameroon

**Kim Bridle**

Australia

**Guy Brock**

USA

**Julie Brown**

New Zealand

**Lindsay Brown**

Australia

**Iguatemy Lourenço Brunetti**

Brazil

**Angela Bruzzaniti**

USA

**Diego Bucci**

Italy

**Steven Budsberg**

USA

**Arndt Buessing**

Germany

**Latifa Bulbul**

Bangladesh

**Silvia Burlina**

Italy

**Andrea Cabarkapa**

Serbia

**Célia Cabral**

Portugal

**Jing Cai**

China

**Shi-Ying Cai**

USA

**Gul Sinem Cakir**

Turkey

**Gioacchino Calapai**

Italy

**Jose Camara**

Portugal

**David Cameron-Smith**

New Zealand

**John Cannell**

USA

**Hongbo Cao**

China

**Huijuan Cao**

China

**Raffaele Capasso**

Italy

**Davie Cappoen**

Belgium

**Laura Carim Todd**

USA

**Klaudija Carovic-Stanko**

Croatia

**Judith Carroll**

USA

**Steven Cartwright**

United Kingdom

**Tina Cartwright**

United Kingdom

**John Cavanagh**

USA

**Carlos Ccavaleiro**

Portugal

**Ali Osman Ceribasi**

Turkey

**Rainer Cermak**

Germany

**Gayathri Chadalapaka**

USA

**Han Chae**

South Korea

**Younbyoung Chae**

South Korea

**Yubo Chai**

USA

**Kelvin Chan**

Hong Kong

**Yin-Ching Chan**

Taiwan

**Chanpen Chanchao**

Thailand

**Phool Chandra**

India

**Anish Chandy**

Malaysia

**Cheng-Shyong Chang**

Taiwan

**Fang-Rong Chang**

Taiwan

**Hsiao-Yun Chang**

Taiwan

**Hsueh-Wei Chang**

Taiwan

**Lee-Tian Chang**

Taiwan

**Mei Ying Chang**

Taiwan

**Shih-Liang Chang**

Taiwan

**Sungwon Chang**

Australia

**Weizhong Chang**

USA

**Ling Changquan**

China

**Pi Yu Chao**

Taiwan

**Srinivasa Chary**

South Africa

**Varanuj Chatsudthipong**

Thailand

**Farid Chemat**

France

**Wei Chen**

China

**Ching-Hsein Chen**

Taiwan

**Pei-Jie Chen**

China

**Yeng Chen**

Malaysia

**Chun-Jen Chen**

Taiwan

**Jing-Hsien Chen**

Taiwan

**Qingyong Chen**

China

**Fang-Pey Chen**

Taiwan

**Hubiao Chen**

Hong Kong

**Henian Chen**

USA

**Jiachun Chen**

China

**Jing Chen**

USA

**Jih-Jung Chen**

Taiwan

**Jaw-Wen Chen**

Taiwan

**Jianxin Chen**

China

**Pei-Ni Chen**

Taiwan

**Shih-Shun Chen**

Taiwan

**Lixin Chen**

China

**Xiuping Chen**

Macao

**Yangchao Chen**

Hong Kong

**Yu-Chun Chen**

Taiwan

**Chin-Yi Cheng**

Taiwan

**Juei-Tang Cheng**

Taiwan

**Jae Hoon Cheong**

South Korea

**Hsiu-Mei Chiang**

Taiwan

**Lucas Chibli**

Brazil

**Shih-Chang Chien**

Taiwan

**Jin Han Chin**

Malaysia

**Siewmooi Ching**

Malaysia

**Salvatore Chirumbolo**

Italy

**Salvatore Chirumbolo**

Italy

**Chien Chih Chiu**

Taiwan

**Wanchun Chiu**

Taiwan

**Hye-Youn Cho**

USA

**William Cs Cho**

Hong Kong

**Won-Kyung Cho**

South Korea

**Yung Hyun Choi**

South Korea

**Sun-Mi Choi**

South Korea

**Shahabuddin Choudhuri**

Bangladesh

**Altino Choupina**

Portugal

**Nho Chu Won**

South Korea

**Lee Suan Chua**

Malaysia

**Anderly Chueh**

Australia

**Jiang Chunming**

China

**Martin Church**

United Kingdom

**Sasitorn Chusri**

Thailand

**Richard Cimanga Kanyanga**

Belgium

**Sofie Claerhout**

Belgium

**Sofie Clais**

Belgium

**Natalie Clark**

USA

**Yuri Clement**

Trinidad and Tobago

**Ignea Codruta**

Greece

**Steven Cok**

USA

**Talita Colomeu**

Brazil

**Violeta Colova-Tsolova**

USA

**Kieran Cooley**

Canada

**Edwin Cooper**

USA

**Werner Cordier**

South Africa

**Paola Costelli**

Italy

**Samantha Coulson**

Australia

**Virginia Cowen**

USA

**Holger Cramer**

Germany

**Helen Cramer**

United Kingdom

**Sybil Crawford**

USA

**Duncan Cromarty**

South Africa

**S Nicole Culos-Reed**

Canada

**Ray Cursons**

New Zealand

**Salvatore Cuzzocrea**

Italy

**Ademar Alves Da Silva Filho**

Brazil

**Sanja Dabelic**

Croatia

**Fulvio D'Acquisto**

United Kingdom

**Christopher D'Adamo**

USA

**Ning Dai**

China

**Andrew Dainty**

United Kingdom

**Abdulkarim Dakah**

Syria

**Archana Damle**

India

**Robert Dantzer**

USA

**Christophe Dardonville**

Spain

**Dana Darwish**

Jordan

**Undurti Das**

USA

**Alok Das Mohapatra**

USA

**Gouri Kumar Dash**

Malaysia

**Matheus Augusto De Bittencourt Pasquali**

Canada

**Solange Lisboa De Castro**

Brazil

**Vincenzo De Feo**

Italy

**Jillian De Gezelle**

USA

**Harry De Koning**

United Kingdom

**Giuseppina De Petro**

Italy

**Damião De Sousa**

Brazil

**Lokesh Deb**

India

**Arnaud Dechamps**

Indonesia

**Felipe De-Faria**

Brazil

**Sukanya Dej-Adisai**

Thailand

**Margarida Delgado**

Portugal

**Roxana Delgado**

USA

**Preeti Desai**

India

**Rahul Deshmukh**

USA

**Serawit Deyno**

Ethiopia

**Suranganie Dharmawardhane**

USA

**Roberta Di Pietro**

Italy

**Teresa Dias**

Portugal

**Konstantinos Dimas**

Greece

**Laurence Dinan**

United Kingdom

**Pei-Hui Ding**

China

**Mohamed Dkhil**

Saudi Arabia

**Jingcheng Dong**

China

**Anton Dormer**

USA

**Patrick Dougherty**

USA

**Jurgen Drewe**

Switzerland

**Donna M Dryden**

Canada

**Dongshu Du**

China

**Jin Ao Duan**

China

**Nawal Dubey**

India

**Pierre Duez**

Belgium

**Heather Duncan**

USA

**Neil Duncan**

South Africa

**Veeramuthu Duraipandiyan**

India

**Elisângela Düsman**

Brazil

**Som Dutt**

India

**Abirlal Dutta**

India

**Chandradhar Dwivedi**

USA

**Gary Dykes**

Malaysia

**Jean Paul Dzoyem**

Cameroon

**Mohammad Ali Ebrahimzadeh**

Iran

**Padilla Eduardo**

Mexico

**Thomas Efferth**

Germany

**Brandon Eggleston**

USA

**Günter Eisele**

Switzerland

**Daisuke Ekuni**

Japan

**Rajaa El Bekay**

Spain

**Mohamed El Gendy**

Canada

**Sabry El-Bahr**

Egypt

**Fatma El-Demerdash**

Egypt

**Esam Elgorashi**

South Africa

**Ishaku Elisha**

South Africa

**Khaled El-Massry**

Egypt

**Nguelefack-Mbuyo Pami Elvine**

Cameroon

**Jurgen Engele**

Germany

**Ephrem Engidawork**

Ethiopia

**Shin Enosawa**

Japan

**Wan Kyu Eo**

South Korea

**Gokhan Eraslan**

Turkey

**Edzard Ernst**

United Kingdom

**Esra Eroglu Ozkan**

Turkey

**S Pelin Ertürküner**

Turkey

**Olorunfemi Eseyin**

Nigeria

**Manuel Estrada**

Chile

**Bruno Eto**

France

**Adaobi Ezike**

Nigeria

**Lucia Helena Faccioli**

Brazil

**Enrico Facco**

Italy

**Saudat Fadeyi**

USA

**Lihong Fan**

China

**Junming Fan**

China

**Yongjun Fan**

Australia

**Chee-Mun Fang**

Malaysia

**Jianqiao Fang**

China

**Jiliang Fang**

China

**Yaghoob Farbood**

Iran

**Eszter Farkas**

Hungary

**Tahereh Farkhondeh**

Iran

**Elham Farsi**

Malaysia

**Sheikh Fayaz Ahmad**

Saudi Arabia

**Bibi Sedigheh Fazly Bazzaz**

Iran

**Qin Feng**

China

**Liang Feng**

China

**Maria Helena Fernandes**

Portugal

**Patricia Fernandes**

Brazil

**Matheus F Fernandes-Pedrosa**

Brazil

**Lydia Ferrara**

Italy

**Vania Ferreira**

Brazil

**Kathryn Field**

Australia

**Theresa Filtz**

Afghanistan

**Susana Fiorentino**

Colombia

**Tatiana Fiuza**

Brazil

**Francisca Cléa Florenço De Sousa**

Brazil

**Andrew Flower**

United Kingdom

**William Folk**

USA

**Gert Folkerts**

Netherlands

**Vinjar Fonnebo**

Norway

**Maria José Fonseca**

Brazil

**Moradali Fouladvand**

Iran

**Jeferson Franco**

Brazil

**Alberte Fransis**

Estonia

**Brian Freed**

USA

**Jan Friedrich**

Canada

**Marcio Fronza**

Brazil

**Reginald Frye**

USA

**Richard Frye**

USA

**Junsheng Fu**

USA

**Jorge Luis Fuentes Lorenzo**

Colombia

**Masaki Fujita**

Japan

**Yohji Fukazawa**

Japan

**Hidefumi Fukumitsu**

Japan

**Isabelle Gaboury**

Canada

**Sudesh Gaidhani**

India

**Peter Gal**

Slovakia

**Angel S Galabo**v

Bulgaria

**Shanmugasundaram Ganapathy-Kanniappan**

USA

**Nitin Gandhi**

India

**Zhan-Guo Gao**

USA

**Zhuye Gao**

China

**Rosa Garcia**

Mexico

**Adriana Garibay-Escobar**

Mexico

**Karunakaran Gauthaman**

India

**Andreea Geamanu**

USA

**Po-Wu Gean**

Taiwan

**Maria Concetta Geloso**

Italy

**Abdella Gemechu**

Ethiopia

**Patricia Gerbarg**

USA

**Kashif Ghafoor**

Saudi Arabia

**Ali Ghasemzadeh**

Malaysia

**Saroj Ghaskadbi**

India

**Irfan Ahmad Ghazi**

India

**Ahmad Ghorbani**

Iran

**Jyotirmoy Ghosh**

India

**Dimitris Giakoustidis**

Greece

**Jan Gielis**

Belgium

**Anwar-Ul-Hassan Gilani**

Ethiopia

**Goezde Girgin**

Turkey

**Bupesh Giri**

India

**Anjan Giriraju**

India

**Erika Gisel**

Canada

**Andy Göbel**

Germany

**Amanuel Godebo**

Ethiopia

**Bhomik Goel**

India

**Antony Gomes**

India

**Nian Gong**

USA

**Zuojiong Gong**

China

**Marie-Paule Gonthier**

France

**Margarete Goppelt-Struebe**

Germany

**Johanna Gostner**

Austria

**Kazuo Goto**

Japan

**Lingshan Gou**

China

**Eman Gouda**

Egypt

**Mary Grace**

USA

**Sandra Grace**

Australia

**Felix Grases**

Spain

**Christopher Gray**

Canada

**Sara Greay**

Australia

**Joel Greenberger**

USA

**Phillip Greenspan**

USA

**Jeffrey Greeson**

USA

**Erik Groessl**

USA

**Amelia Guadalupe-Grau**

Denmark

**Joao Bosco Guerreiro Da Silva**

Brazil

**Corina Guethlin**

Germany

**Gunjan Guha**

USA

**Davide Guido**

Italy

**Gaël Guilhem**

France

**Ilhami Gulcin**

Turkey

**Iram Gull**

Pakistan

**Songxue Guo**

China

**Xinfeng Guo**

China

**Feifan Guo**

China

**Zhi-Ling Guo**

USA

**Krishna Gupta**

India

**Rajiv Gupta**

India

**Saurabh Gupta**

India

**Kitae Ha**

South Korea

**Ki-Chan Ha**

South Korea

**Marwan Habiba**

United Kingdom

**Marshall Hagins**

USA

**Heidemarie Haller**

Germany

**Khaled Hamden**

Tunisia

**Nadia Hamdy**

Egypt

**Ann Hamilton**

USA

**Richard Hammerschlag**

USA

**Jing-Yan Han**

China

**Sangmi Han**

South Korea

**Sang-Bae Han**

South Korea

**Sung Soo Han**

South Korea

**Manjunatha Hanumanthappa**

India

**Hideaki Hara**

Japan

**Ulf Harnack**

Germany

**Maryam Hassanzadeh Bashtian**

Iran

**Cheryl Hawk**

USA

**Dongyuan He**

China

**Jun He**

China

**Lang He**

China

**Ulla Hedegaard**

Denmark

**Jodi Hedges**

USA

**Horacio Heinzen**

Uruguay

**S Hemalatha**

India

**Ali Asghar Hemmati**

Iran

**Rini Hendriani**

Indonesia

**Ana Henriques**

Portugal

**Maria Das Graças Henriques**

Brazil

**Alberto Marcos Heredia Rizo**

Spain

**Mona Hetta**

Egypt

**Koji Higai**

Japan

**Clélia Akiko Hiruma-Lima**

Brazil

**Joanna Hlebowicz**

Sweden

**Johanna Hok**

Sweden

**Richard Holubkov**

USA

**Kam-Lun Ellis Hon**

Hong Kong

**Jin Tae Hong**

South Korea

**Mee Young Hong**

USA

**Mahmoud Hossein**i

Iran

**Hossein Hosseinzadeh**

Iran

**Wen-Chi Hou**

Taiwan

**Yih-Shou Hsieh**

Taiwan

**Hsin Hsiu**

Taiwan

**Shih-Lan Hsu**

Taiwan

**Jun-Te Hsu**

Taiwan

**Zhi-Qi Hu**

China

**Dongsheng Hu**

China

**Jiang Hu**

China

**Yingwei Hu**

China

**Yuanjie Hu**

USA

**Cheng-Yang Huang**

Taiwan

**Yi-Tsau Huang**

Taiwan

**Jinhai Huang**

China

**Kun Huang**

USA

**Mei-Hua Huang**

USA

**Shou-Hsien Huang**

Taiwan

**Wei Huang**

USA

**Xiaoxing Huang**

China

**Sona Hudecova**

Slovakia

**Jutta Huebner**

Germany

**John Hughes**

United Kingdom

**Mahmoud Huleihel**

Israel

**Lee Hullender Rubin**

USA

**Laith Hussain-Alkhateeb**

Sweden

**Minwoo Hwang**

South Korea

**Shinn-Jang Hwang**

Taiwan

**Jin Won Hyun**

South Korea

**Zein Ibrahim**

Egypt

**Savarimuthu Ignacimuthu**

India

**Zullies Ikawati**

Indonesia

**Mohammad Zafar Imam**

Bangladesh

**Paulo Imamura**

Brazil

**Naoki Inagaki**

Japan

**Masatoshi Inden**

Japan

**Zafar Iqbal**

Pakistan

**Mehrdad Iranshahi**

Iran

**Yoshitaka Isaka**

Japan

**Liz Isenring**

Australia

**Amirul Islam**

Bangladesh

**Md Rafikul Islam**

Bangladesh

**Sabariah Ismail**

Malaysia

**Toshinori Ito**

Japan

**Kazuhisa Iwabuchi**

Japan

**Ezekiel Olugbenga Iwalewa**

Nigeria

**Vidhya Iyer**

USA

**Mohamed Jaabir**

India

**Eric Jacobson**

USA

**Yves Jacquot**

France

**Hartmut Jaeschke**

USA

**Sediqeh Jalali**

Iran

**Naveena Janakiram**

USA

**Hae-Dong Jang**

South Korea

**Su Hee Jang**

South Korea

**Insoo Jang**

South Korea

**Tae Jung Jang**

South Korea

**Upali W Jayasinghe**

Australia

**Kamalan Jeevaratnam**

Malaysia

**Kristina Jenett-Siems**

Germany

**Myung-Shin Jeon**

South Korea

**Daewon Jeong**

South Korea

**Woo-Sik Jeong**

South Korea

**Minjeong Jeong**

South Korea

**Rajesh Kumar Jha**

India

**Guang Ji**

China

**Lin-Tao Jia**

China

**Yantao Jia**

China

**Han Jia**

China

**Hong Jiang**

China

**Weihua Jiang**

USA

**Meiyan Jiang**

USA

**Ligang Jie**

China

**Maria Adelina Jiménez-Arellanes**

Mexico

**Chengyun Jin**

China

**Tomoko Jippo**

Japan

**Stefanie Joos**

Germany

**Amita Joshi**

India

**Chintamani Joshi**

India

**Narendra Joshi**

India

**Swati Joshi-Barve**

USA

**Il Lae Jung**

South Korea

**Helgi Jung**

Mexico

**Suh-Hang Juo**

Taiwan

**Wan Jy**

China

**Shuk-Man Ka**

Taiwan

**Satyan Kalkunte**

USA

**Venkatesh Kamath Kamath**

India

**Yuto Kamei**

Japan

**Bernard Kamsu Foguem**

France

**Tae Jin Kang**

South Korea

**Ning Kang**

China

**Hee-Kyoung Kang**

South Korea

**Young-Hee Kang**

South Korea

**Yu-Hsun Kao**

Taiwan

**Panayotis Karayannakidis**

Greece

**David Katerere**

South Africa

**Ruchika Kaul-Ghanekar**

India

**Tejinder Kaur**

India

**Srini Kaveri**

France

**Eric Kelley**

USA

**William Kell**y

New Zealand

**Moira Kelly**

United Kingdom

**Deborah Kennedy**

Canada

**Janet Kern**

USA

**Paula Kersten**

New Zealand

**Rana Keyhanmanesh**

Iran

**Mohamed Khalil**

Saudi Arabia

**Murad Khan**

Pakistan

**Haroon Khan**

Pakistan

**Kainat Khan**

India

**Mohd Khan**

South Africa

**Muhammad Khan**

Pakistan

**Zakia Khanam**

Malaysia

**Mahnaz Khanavi**

Iran

**Alfi Khatib**

Malaysia

**Mahmoud Khattab**

Egypt

**Mohamed T Khayyal**

Egypt

**Elian Khazneh**

Czech Republic

**Nafiseh Khosravi**

Iran

**Ar Khuda-Bukhsh**

India

**Lik Voon Kiew**

Malaysia

**Sharon Kilbreath**

Australia

**Dae-Ok Kim**

South Korea

**Hyung Sik Kim**

South Korea

**Hyeung-Rak Kim**

South Korea

**Hee Young Kim**

South Korea

**Hyunho Kim**

South Korea

**Hyun Jik Kim**

South Korea

**Won Ho Kim**

South Korea

**Ji Yeon Kim**

South Korea

**Joong-Sun Kim**

South Korea

**Jae Yong Kim**

South Korea

**Hojun Kim**

South Korea

**Jong Bin Kim**

South Korea

**Young-Min Kim**

South Korea

**Kun Hyung Kim**

South Korea

**Sun Kwang Kim**

South Korea

**Sung Kim**

South Korea

**Jong-Choon Kim**

South Korea

**Wook Kim**

South Korea

**Young-Cheul Kim**

USA

**Shintaro Kinugawa**

Japan

**Roman Kireev**

Spain

**Irina Kiseleva**

Russian Federation

**Navneet Kishore**

South Africa

**Anoop Kishore**

India

**Attila Kiss**

Sweden

**Soressa Kitessa**

Australia

**Nadja Klafke**

Germany

**Riana Kleynhans**

South Africa

**Helmut Klocker**

Austria

**Chun Hay Ko**

Hong Kong

**Kwangsuk Ko**

South Korea

**Biplob Koch**

India

**George Koffuor**

Ghana

**Itaru Kojima**

Japan

**Yoshihiro Komada**

Japan

**Michio Komai**

Japan

**Sureshbabu Kondaveeti**

India

**Ah-Ng Tony Kong**

USA

**Xiangying Kong**

China

**Sumet Kongkiatpaiboon**

Thailand

**Alice Kongsted**

Denmark

**Rituraj Konwar**

India

**Yun Hyung Koog**

South Korea

**Shigeru Kotake**

Japan

**Demetrios Kouretas**

Greece

**Stanisław Kowalski**

Poland

**Omer Koz**

Turkey

**Rajesh Kp**

India

**Sandra Kraljevic Pavelic**

Croatia

**Nataly Kravchenko-Balasha**

Israel

**Nandakumar Krishnadas**

India

**Agnete Egilsdatter Kristoffersen**

Norway

**Anil Kumar**

India

**Akhilesh Kumar**

India

**Amit Kumar**

India

**Nitesh Kumar**

India

**Vikas Kumar**

India

**Rajesh Kumar**

India

**Dinesh Kumar**

India

**Ravi Kumara**

India

J**oydeb Kumar Kundu**

Bangladesh

**Vijayanarayana Kunhikatta**

India

**Kazuhiro Kunimasa**

Japan

**Ramkumar Kunka Mohanram**

India

**Ajaikumar Kunnumakkara**

USA

**Jinn Rung Kuo**

Taiwan

**Sajeera Kupittayanant**

Thailand

**Hokeun Kwon**

USA

**Hokeun Kwon**

USA

**Jung-Taek Kwon**

South Korea

**Dong-Yeul Kwon**

South Korea

**Umar Kyari Sandabe**

Nigeria

**Giovanni Michele Lacalandra**

Italy

**Sara Ladu**

Italy

**Rene Lafont**

France

**Joao Lago**

Brazil

**Vicente Lahera**

Spain

**Lily Lai**

United Kingdom

**Jung Nien Lai**

Taiwan

**Kuang-Chi Lai**

Taiwan

**Namrita Lall**

South Africa

**Fanny Lalot**

Switzerland

**Ouattara Lamoussa Paul**

Burkina Faso

**Jean Langlois**

France

**George Lansner**

USA

**Li Xing Lao**

Hong Kong

**Malinee Laopaiboon**

Thailand

**Gee W Lau**

USA

**Nico Laubscher**

South Africa

**Romy Lauche**

Germany

**Horng-Liang Lay**

Taiwan

**Diamanto Lazari**

Greece

**Bombi Lee**

South Korea

**Meeyoung Lee**

South Korea

**Myeong Soo Lee**

South Korea

**Hyangsook Lee**

South Korea

**Jang-Hern Lee**

South Korea

**Tzung-Yan Lee**

Taiwan

**Kwang Ho Lee**

South Korea

**Soo Young Lee**

South Korea

**Huei-Jane Lee**

Taiwan

**Ju Ah Lee**

South Korea

**Min Won Lee**

South Korea

**Bong Hyo Lee**

South Korea

**Sanghoon Lee**

South Korea

**Chelsey Lemaster**

USA

**Wossenseged Lemma**

Ethiopia

**Ping-Chung Leung**

Hong Kong

**George Lewith**

United Kingdom

**Juan Li**

China

**Jie Li**

China

**Jing Li**

China

**Hu Li**

USA

**Chuanfu Li**

China

**Chenrui Li**

China

**Kewei Li**

China

**Li Li**

USA

**Lingyun Li**

China

**Min Li**

Hong Kong

**Ping Li**

China

**Xuting Li**

China

**Ya-Jun Li**

China

**Yousheng Li**

China

**Yu Li**

China

**Zilong Li**

China

**Cheng-Rui Li**

USA

**Min Li**

USA

**Peng Li**

USA

**Shulin Li**

USA

**Tsai-Chung Li**

Taiwan

**Xin-Min Li**

Canada

**Yin Lian**

China

**Fan-Rong Liang**

China

**Hui-Fen Liao**

Taiwan

**Xing Liao**

United Kingdom

**Byungmook Lim**

South Korea

**Yang Mooi Lim**

Malaysia

**Diingbo Lin**

USA

**Yin-Ku Lin**

Taiwan

**Annemette Bondo Lind**

Denmark

**Ulrike Lindequist**

Germany

**Ingrid Liodden**

Norway

**Ying-Ming Liou**

Taiwan

**Patricia Lisboa**

Brazil

**Alyson Littman**

USA

**Jun Liu**

China

**Chenghai Liu**

China

**Huiqing Liu**

USA

**Jianping Liu**

China

**Cun-Zhi Liu**

China

**Bo Liu**

China

**Hongbing Liu**

China

**Xusheng Liu**

China

**Quanhong Liu**

China

**Shuman Liu**

China

**Yi-Wen Liu**

Taiwan

**Richard Lobo**

India

**Dario Lofrumento**

Italy

**Carl Lombard**

South Africa

**Suguna Lonchin**

India

**Andrew Long**

United Kingdom

**Angela Lopes**

Brazil

**Guillermo Lopez**

Mexico

**Nattaya Lourith**

Thailand

**Brenda Lovell**

Canada

**Zhigang Lu**

China

**Qing-Yi Lu**

USA

**Weidong Lu**

USA

**Larissa Lucena Périco**

Brazil

**Iréne Lund**

Sweden

**Jian Luo**

USA

**Deyan Luo**

China

**Devaraj M**

India

**Choong Je Ma**

South Korea

**Chaomei Ma**

China

**Liang Ma**

China

**Bingji Ma**

China

**Ian Mackenzie**

United Kingdom

**Sarah Maddocks**

United Kingdom

**Lizandra Magalhães**

Brazil

**Pérola Magalhães**

Brazil

**Pamela Maher**

USA

**Narayan Maheswary**

Bangladesh

**Ayman Mahmoud**

Egypt

**Fawzi Mahomoodally**

Mauritius

**Cristiane Maia**

Brazil

**Arindam Maitra**

India

**Juraj Majtan**

Slovakia

**Tshepiso Jan Makhafola**

South Africa

**Suzana Makpol**

Malaysia

**N Malangu**

South Africa

**Tauseef Malik**

India

**Mao-Qiang Man**

USA

**Thamilvaani Manaharan**

Malaysia

**Azadeh Manayi**

Iran

**Mahitosh Mandal**

India

**Soma Mandal**

Canada

**Santi M Mandal**

India

**Melânia Manfron**

Brazil

**Supachoke Mangmool**

Thailand

**Tran Manh Hung**

Viet Nam

**Luigi Manni**

Italy

**Sharif Mansor**

Malaysia

**Jose Mansure**

Canada

**Isabelle Marc**

Canada

**Cristina Marcucci**

Brazil

**Donald Marcus**

USA

**Abdalbasit Mariod**

Sudan

**Jacquelyn Marsh**

Canada

**Wolfgang Marx**

Australia

**Robert Mathie**

United Kingdom

**Vitor Mati**

Brazil

**Ivana Matic**

Serbia

**Sannah Mativandlela**

South Africa

**Hisashi Matsuda**

Japan

**Sithandiwe Mazibuko**

South Africa

**Kishor Mazumder**

Bangladesh

**Karalyn Mcdonald**

Australia

**Lyndy Mcgaw**

South Africa

**Jane Mchowat**

USA

**Ahmed Mediani**

Algeria

**Eugene Megnassan**

Cote d'Ivoire

**Hayelom Mekonen**

Ethiopia

**Lighua Meng**

China

**Zhi-Hong Meng**

China

**Francesca Menniti**

Italy

**Filipe Mergulhao**

Portugal

**Francois Meurens**

Canada

**Marion Meyer**

South Africa

**Rikke Louise Meyer**

Denmark

**Sascha Meyer**

Germany

**Oliver Micke**

Germany

**Maria Miguel**

Portugal

**Marius Mihasan**

Romania

**Jessica Miller**

USA

**Mladen Milos**

Croatia

**Selvaraj Miltonprabu**

India

**Hyeyoung Min**

South Korea

**Do Sik Min**

South Korea

**Junki Min**

South Korea

**Bi Minggang**

China

**Silvano Mior**

Canada

**Maria Leticia Miranda Fernandes Estevinho**

Portugal

**Shingen Misaka**

Japan

**Ashwini Mishra**

USA

**Megha Mittal**

India

**Tempei Miyaji**

Japan

**Shingo Miyata**

Japan

**Shingo Miyata**

Japan

**Wataru Mizunoya**

Japan

**Unnikrishnan Mk**

India

**Andrei Mocan**

Romania

**Reda Mohammed**

Egypt

**Maradumane Mohan**

USA

**Ayaz Mohd**

Oman

**Nurul 'Izzah Mohd Sarmin**

Malaysia

**Yasmin Anum Mohd Yusof**

Malaysia

**Alex Molassiotis**

Hong Kong

**Fernando P Molina-Heredia**

Spain

**Marisa Moller Wolmarans**

South Africa

**Robson Monteiro**

Brazil

**Lianet Monzote**

Cuba

**Michael Moore**

United Kingdom

**Nicholas Moore**

France

**Alex Moroz**

USA

**Jane Morrell**

Sweden

**Mohamed Morsy**

Egypt

**Said Moselhy**

Saudi Arabia

**Abdel-Tawab Mossa**

Egypt

**Ramzi Mothana**

Saudi Arabia

**Andrea Motoyama**

Brazil

**Andrea Motta**

Italy

**Raymond Simplice Mouokeu**

Cameroon

**Mack Moyo**

South Africa

**Jayesh Mudgal**

India

**Karsten Muenstedt**

Germany

**Aliyu Muhammad**

Nigeria

**Naveed Muhammad**

Pakistan

**Faqir Muhammad**

USA

**Kalpana Mujoo**

USA

**Lillian Mukandiwa**

South Africa

**Rofhiwa Bridget Mulaudzi**

South Africa

**Eshetu Mulisa**

Ethiopia

**Niki Munk**

USA

**Renuka Munshi**

India

**Akira Murakami**

Japan

**Tsugiya Murayama**

Japan

**Frauke Musial**

Norway

**Michihiro Mutoh**

Japan

**Peter Mwitari**

Kenya

**Chang-Seon Myung**

South Korea

**Mahadevan N**

India

**Kesara Na-Bangchang**

Thailand

**Seyed Mohammad Nabavi**

Iran

**Pratibha Nadig**

India

**Richard J Naftalin**

United Kingdom

**Raghuram Nagarathna**

India

**Basavraj Nagoba**

India

**Vinny Naidoo**

South Africa

**Umberin Najeeb**

Canada

**Toshiaki Nakano**

Taiwan

**Ayse Nalbantsoy**

Turkey

**Donghyun Nam**

South Korea

**Kim Nam-Hee**

South Korea

**Madhavan Nampoothiri**

India

**Betty Namuddu**

Uganda

**Mohsen Naseri**

Iran

**Andrés Navarrete**

Mexico

**Yogendra Nayak**

India

**Yogendra Nayak**

India

**Ashwell Ndhlala**

South Africa

**Dieudonne Ndjonka**

Cameroon

**Candace Necyk**

Canada

**Bimla Nehru**

India

**Fatemeh Nejatzadeh**

Iran

**Telesphore Benoit Nguelefack**

Cameroon

**Endalkachew Nibret**

Ethiopia

**Glenda Nicioli Da Silva**

Brazil

**Loredan Stefan Niculescu**

Romania

**Akio Niimi**

Japan

**Nik Mohd Afizan Nik Abd. Rahman**

Malaysia

**Cristina Nogueira**

Brazil

**Fabiana Nonato**

Brazil

**Cesar Nopo-Olazabal**

USA

**Hedvig Nordeng**

Norway

**Farah Noureen**

Pakistan

**Maja Nowakowski**

USA

**Fidele Ntie-Kang**

Cameroon

**Lorena Nunez**

South Africa

**Mohd Fahami Nur Azlina**

Malaysia

**Armania Nurdin**

Malaysia

**Olawole Obembe**

Nigeria

**Erica Oberg**

USA

**Kylie O'Brien**

Australia

**Young Ock Kim**

South Korea

**José-Enrique O'Connor**

Spain

**Olabisi Oduwole**

Nigeria

**Junetsu Ogasawara**

Japan

**Haruko Ogawa**

Japan

**Adenike Ogunshe**

Nigeria

**Oluwafemi Oguntibeju**

South Africa

**Byeongsang Oh**

Australia

**Myung Sook Oh**

South Korea

**Shreesh Ojha**

United Arab Emirates

**Yoshihiro Okamoto**

Japan

**Charles Okoli**

Nigeria

**Marina Okoshi**

Brazil

**Theophine Okoye**

Nigeria

**Tadayoshi Okumura**

Japan

**Toshikatsu Okumura**

Japan

**Olumayokun Olajide**

United Kingdom

**Oyinlola Olaokun**

South Africa

**Bogdan Olenyuk**

USA

**Maira Souza Oliveira**

Brazil

**Horacio Olivo**

USA

**Edwin Omeje**

Nigeria

**Tonia Onyeka**

Nigeria

**Nor Hayati Othman**

Malaysia

**Bamidele Owoyele**

Nigeria

**Ademola Oyagbemi**

Nigeria

**Marcin Ozarowski**

Poland

**Yoko Ozawa**

Japan

**Cristina Pacheco Soares**

Brazil

**Rabindra Padhy**

India

**Alessio Paffoni**

Italy

**Aravinda Pai**

India

**Poungrat Pakdeechote**

Thailand

**Shanthi Palanivelu**

India

**Sarawoot Palipoch**

Thailand

**Fabricio Pamplona**

Brazil

**Tai-Long Pan**

Taiwan

**Abhay K. Pandey**

India

**Siyaram Pandey**

Canada

**Perumal Pandurangan**

India

**Jong-Hwei Pang**

Taiwan

**Xiufeng Pang**

China

**Manju Panghal**

India

**Li-Heng Pao**

Taiwan

**Wansu Park**

South Korea

**Heonyong Park**

South Korea

**Jong Park**

USA

**Jongbae Park**

USA

**Kye Won Park**

South Korea

**Chan-Hong Park**

South Korea

**Seong-Uk Park**

South Korea

**Yongsoon Park**

South Korea

**Donna Parker**

USA

**Kyung-Hyun Park-Min**

USA

**Visweswara Rao Pasupuleti**

Malaysia

**Vinood Patek**

United Kingdom

**Paras Patel**

India

**Ravi Patel**

USA

**Charlotte Paterson**

United Kingdom

**Ashish Pathak**

USA

**Srinivas Patnala**

South Africa

**Alexandra Paulo**

Portugal

**Maxime Pellegrin**

Switzerland

**Wenbo Peng**

Australia

**Philip Peplow**

New Zealand

**Andrés Pereañez**

Colombia

**Rosa M Perez-Gutierrez**

Mexico

**Cynthia Peterson**

Switzerland

**Kenneth Pfarr**

Germany

**Ngoc Minh Pham**

Viet Nam

**Mallikarjuna Rao Pichika**

Malaysia

**Anatole Constant Pieme**

Cameroon

**Karen Pilkington**

United Kingdom

**Shubhangi Pingle**

India

**Somchai Pinlaor**

Thailand

**Fabio Pittella Silva**

Brazil

**Marcia Regina Piuvezam**

Brazil

**Maribel Plascencia-Jatomea**

Mexico

**Adrian Martin Pohlit**

Brazil

**Bernard Poitevin**

France

**Ganesan Ponesakki**

India

**Beaudelaire Ponou Kemvoufo**

Cameroon

**Antony Porcino**

Canada

**Alberto Porta**

Italy

**Fernanda Portaro**

Brazil

**Jalal Pourahmad**

Iran

**Spyros Pournaras**

Greece

**Kirti Prabhu**

India

**John Preshanth Kumar**

India

**Matthias Preusser**

Austria

**Sarah Price**

United Kingdom

**Gerhard Prinsloo**

South Africa

**Long Qu**

USA

**Sara Quandt**

USA

**Cassandra Quave**

USA

**Annie Quignard-Boulange**

France

**Jael Quintero**

Mexico

**Raymond Quock**

USA

**Rahmatullah Qureshi**

Pakistan

**Karim Raafat**

Lebanon

**Mohamed Rabeh**

Egypt

**Gandhi Radis-Baptista**

Brazil

**Atiar Rahman**

Bangladesh

**Md Masudur Rahman**

Bangladesh

**Najm-Ur Rahman**

Pakistan

**Batool Rahmati**

Iran

**Mohammad Rahmati Yamchi**

Iran

**Mohammed Rahmatullah**

Bangladesh

**Revathi Rajappan**

India

**Nutan Rajput**

India

**Harinantenaina Rakotondraibe**

USA

**Aswatha Ram H.N.**

India

**Venkatesha Perumal Ramachandran**

India

**Vadde Ramakrishna**

India

**Ravindhran Ramalingam**

India

**Surash Ramanathan**

Malaysia

**Sujatha Ramasamy**

Malaysia

**Radhakrishnan Rameshkumar**

India

**Sonia Ramos**

Spain

**Anand Ramteke**

India

**Ruiqiong Ran**

USA

**Chandana Rao**

India

**Iraj Rasooli**

Iran

**Zahid Rasul**

France

**Sudipt Rath**

India

**Edward Ratovitski**

USA

**Jo-Anne Rayner**

Australia

**Daniel Redwood**

USA

**Ralf Regenthal**

Germany

**Juliana Quero Reimão**

Brazil

**Thomas Reinhold**

Germany

**Flavia Aparecida Resende**

Brazil

**Morayma Reyes**

USA

**Jennifer Rioux**

USA

**Cheryl Ritenbaugh**

USA

**Syed Ibrahim Rizvi**

India

**Pierre-Yves Rodondi**

Switzerland

**Juan Francisco Rodríguez-Landa**

Mexico

**Victor Romanov**

USA

**Irma Romero**

Mexico

**Philippe Rondeau**

France

**Ruiming Rong**

China

**Pornpimol Rongnoparut**

Thailand

**Elaine Rosas**

Brazil

**Ema Rosas-Burgos**

Mexico

**Bjorn Rosengren**

Sweden

**Rina Rosin-Arbesfeld**

Israel

**Anastasia Rowland-Seymour**

USA

**Aditi Roy**

India

**Abdur Rub**

India

**Eduardo Ruiz-Bustos**

Mexico

**Bernhard Ryffel**

France

**Jong Hoon Ryu**

South Korea

**Bashar Saad**

Israel

**Niina Saarinen**

Finland

**Maria Jose Saavedra**

Portugal

**Masoumeh Sabetkasaei**

Iran

**Katia Sabino**

Brazil

**Sherry Sachdeva**

India

**Azmi Johari Saiful**

Malaysia

**M Ram Sairam**

Canada

**Cagri Sakalar**

Turkey

**Meena Sakharkar**

Japan

**Hossam Salama**

Egypt

**Rciardo Salazar**

Mexico

**Sabita Saldanha**

USA

**Ammar Saleem**

Canada

**Avni Sali**

Australia

**Avni Sali**

Australia

**Naguib Salleh**

Malaysia

**Jagdeep Sandhu**

Canada

**Louis Pergaud Sandjo**

Cameroon

**Senthil Kumar Sankareswaran**

India

**Enrica Santarcangelo**

Italy

**Juan F Santibanez**

Serbia

**Rogerio Saraiva**

Brazil

**Sudarsini Saravanabhavan**

Saudi Arabia

**Moklesur Sarker**

Japan

**Bruno Sarmento**

Portugal

**Jerome Sarris**

Australia

**Masa Sasagawa**

USA

**Jiichiro Sasaki**

Japan

**Sreenivasan Sasidharan**

Malaysia

**Hiromi Sato**

Japan

**Neeraj Saxena**

USA

**Donna Scarborough**

USA

**Elad Schiff**

Israel

**Sallie Schneider**

USA

**Dominik Schöndorf**

Germany

**Robert Schulman**

USA

**Elita Scio**

Brazil

**Marília Seelaender**

Brazil

**Shuichi Segawa**

Japan

**Veronique Seidel**

United Kingdom

**Shamala Devi Sekaran**

Malaysia

**Sonal Sekhar**

India

**Samy Selim**

Egypt

**Jasmine Selvakumar**

India

**Jonathan Sembrano**

USA

**David Senchina**

USA

**Chaminda Jayampath Seneviratne**

Singapore

**Surojeet Sengupta**

USA

**Dong-Wan Seo**

South Korea

**Byung-Kwan Seo**

South Korea

**Sonia Sequeira**

USA

**Jadranka Sertic**

Croatia

**Ben Sessa**

United Kingdom

**Robert Sewell**

United Kingdom

**Nadia Shaban**

Egypt

**Nermeen Shaffie**

Egypt

**Abdus Saboor Shah**

China

**Zahoor Shah**

USA

**Mohammad Reza Shakibaie**

Iran

**Baoci Shan**

China

**Hongcai Shang**

China

**Muthu Shanmugam**

India

**Jiangang Shen**

Hong Kong

**Xueyong Shen**

China

**Gajanan Sherbet**

United Kingdom

**Dazhuo Shi**

China

**Xianbao Shi**

China

**Workineh Shibeshi**

Ethiopia

**Su Shi-Bing**

China

**Jamil Shilpi**

Bangladesh

**Insop Shim**

South Korea

**Kazunori Shimada**

Japan

**Byung-Cheul Shin**

South Korea

**In Sik Shin**

South Korea

**Thimmappa Shivanandappa**

India

**Yun Hee Shon**

South Korea

**Mohamed Shoukri**

Saudi Arabia

**Yukihiro Shoyama**

Japan

**Richa Shri**

India

**Shruti Shukla**

South Korea

**Ines Sifaoui**

Tunisia

**Parames Sil**

India

**Teresinha Silva**

Brazil

**Edgar Silveira**

Brazil

**Ana Paula Simões-Wüst**

Switzerland

**Amanda Simons**

USA

**Maree Simpson**

Australia

**Zia-Ud-Din Sindhu**

Pakistan

**Harminder Singh**

India

**Nihar Singh**

India

**Pankaj Singh**

USA

**Rekha Singh**

USA

**Sarman Singh**

India

**Mukeshj Singh**

India

**Neeraj Sinha**

India

**Sivaperumal Sivaramakrishnan**

India

**Dan Sliva**

USA

**Caroline A Smith**

Australia

**Karen Snider**

USA

**Luiz Soares**

Brazil

**Stephanie Sohl**

USA

**Didem Sohretoglu**

Turkey

**Eeva Sointu**

USA

**Rosa Solã**

Spain

**Elizabeth Soliday**

USA

**Maha Soltan**

Egypt

**Rakesh Somani**

India

**Sivagurunathan Somasundaram**

USA

**Avinash Sonawane**

India

**Tuzz-Ying Song**

Taiwan

**Hong-Li Song**

China

**Jianguo Song**

China

**Yiqing Song**

USA

**Germain Sotoing Taïwe**

Cameroon

**Ilavenil Soundharrajan**

South Korea

**Lauro Souza**

Brazil

**Preethi Soysa**

Sri Lanka

**Claire Spears**

USA

**Mariana Spetea**

Austria

**Sarangapani Sreelatha**

Singapore

**Bungorn Sripanidkulchai**

Thailand

**Rungrudee Srisawat**

Thailand

**Anshu Srivastava**

India

**Milan Stankovic**

Serbia

**Amie Steel**

Australia

**Elizabeth Steels**

Australia

**Vanessa Steenkamp**

South Africa

**Marius Stefan**

Romania

**Christopher Stevenson**

Australia

**Joanna Stewart**

New Zealand

**Emilie Stolarczyk**

United Kingdom

**David Stresser**

USA

**Trine Stub**

Norway

**Chun-Li Su**

Taiwan

**Jason Su**

USA

**Ming-Jai Su**

Taiwan

**Chin-Cheng Su**

Taiwan

**Chun-Xiang Su**

China

**Zi-Ren Su**

China

**Alírica Suárez**

Venezuela

**Parasuraman Subramani**

Malaysia

**Mahesh Subramanian**

India

**Sorimuthu Subramanian**

India

**Appian Subramoniam**

India

**Ganapasam Sudhandiran**

India

**Jau-Ling Suen**

Taiwan

**Ivana Suffredini**

Brazil

**Hong-Won Suh**

South Korea

**Hyung Joo Suh**

South Korea

**Yoo-Hun Suh**

South Korea

**Kyoungho Suk**

South Korea

**Shaida Fariza Sulaiman**

Malaysia

**Shubhankar Suman**

USA

**Peng Sun**

USA

**Xuejun Sun**

China

**Tobias Sundberg**

Sweden

**Sugandhika Suresh**

Sri Lanka

**TA Ajith**

India

**Hend Tag**

Egypt

**Reina Taguchi**

Japan

**Reinaldo Takahashi**

Brazil

**Hiroshi Takeda**

Japan

**Kazuhiro Takemoto**

Japan

**Laura Talarico**

Argentina

**Steve Talcott**

USA

**Jean De Dieu Tamokou**

Cameroon

**Hiroyuki Tanaka**

Japan

**Chanderdeep Tandon**

India

**Tao Tang**

China

**Suni Tang**

USA

**Wei Tang**

Japan

**Yong Tang**

China

**Yaxiong Tang**

China

**Yi Tang**

China

**Lijian Tao**

China

**Arda Tasatargil**

Turkey

**Anil Tatiya**

India

**Aldo Tava**

Italy

**Zahra Tayarani-Najaran**

Iran

**Ann Taylor**

USA

**Lisa Taylor-Swanson**

USA

**Gerald Ngo Teke**

Cameroon

**Gerald Ngo Teke**

Cameroon

**Nitin Telang**

USA

**Shirley Telles**

India

**Rémy Bertrand Teponno**

Cameroon

**Masanori Terasaki**

Japan

**Michael Teut**

Germany

**Supinya Tewtrakul**

Thailand

**Nai-Peng Tey**

Malaysia

**Nooruddin Thajuddin**

India

**Lal Thapa**

Nepal

**Kalaivani Thiagarajan**

India

**George Thomson**

United Kingdom

**Derun Tian**

China

**Stephanie Tjen-A-Looi**

USA

**Anil Kumar Tomar**

India

**Omer Toprak**

Turkey

**Masaru Toriyama**

Japan

**Marcio Torsoni**

Brazil

**Santoshkumar Tota**

India

**Oliver Treeck**

Germany

**Debu Tripathy**

USA

**Boryana Trusheva**

Bulgaria

**Fu-Ming Tsai**

Taiwan

**Chin-Chuan Tsai**

Taiwan

**Katrina Tsang**

Hong Kong

**William Tsang**

China

**Karl Wk Tsim**

China

**Pengfei Tu**

China

**Xiang Tu**

China

**Rajkumar Tulsawani**

India

**Shaikh Jamal Uddin**

Bangladesh

**Carlos Ueira-Vieira**

Brazil

**Rizwan Ul Haq**

Pakistan

**Dana Ullman**

USA

**Virgilijus Uloza**

Lithuania

**Jae-Young Um**

South Korea

**Padmaja Vaidyanathan**

India

**Abedin Vakili**

Iran

**Ben Valdez**

USA

**Dora Valencia**

Mexico

**Marieke Van Puymbroeck**

USA

**Jacob Van Tonder**

South Africa

**Sandy Van Vuuren**

South Africa

**Candice Van Wyk**

South Africa

**Robyn Van Zyl**

South Africa

**Ina Vandebroek**

USA

**Luca Vannucci**

Czech Republic

**Seda Vatansever**

Turkey

**Dhiraj Vattem**

USA

**Mahdi Vazirian**

Iran

**Krishna Kumar Veeravalli**

USA

**Ramaprabhu Vempati**

India

**Chandana Venkateswara Rao**

India

**Monika Vishnoi**

USA

**G L Viswanatha**

India

**Luis Vitetta**

Australia

**Jiri Vrba**

Czech Republic

**Vladimir Vuksan**

Canada

**James Wachira**

USA

**Shelli Waetzig**

USA

**Shinya Wakusawa**

Japan

**Ju-Bo Wang**

China

**Bin Wang**

USA

**Ronald Wang**

Hong Kong

**Xiaoge Wang**

China

**Chong-Zhi Wang**

USA

**Fengshan Wang**

China

**Junpeng Wang**

USA

**Jian Wang**

China

**Shu-Ming Wang**

USA

**Shusheng Wang**

USA

**Guangjun Wang**

China

**Xiao Wang**

China

**Xiao-Ning Wang**

China

**Yinye Wang**

China

**Yingwei Wang**

China

**Wei Wang**

USA

**Zhijun Wang**

USA

**Jon Wardle**

Australia

**Narelle Warren**

Australia

**Ramida Watanapokasin**

Thailand

**Pierre Watcho**

Cameroon

**Chatchai Wattanapiromsakul**

Thailand

**John Weber**

Canada

**Laura Weeks**

Canada

**Jiang Wei**

China

**Ying Wei**

China

**Xiaolin Wei**

China

**Kuo-Ching Wen**

Taiwan

**Zhining Wen**

China

**Fu-Qiang Wen**

China

**Chia-Jui Weng**

Taiwan

**Ursula Werneke**

Sweden

**Maria Fernanda Werner**

Brazil

**Janis Whitlock**

USA

**Barbara Wider**

United Kingdom

**C Dilrukshi Wijayarathna**

Sri Lanka

**Jenny Wilkinson**

Australia

**Sonia Will**

Brazil

**Patrick Wilson**

USA

**Ursula Wolf**

Switzerland

**Benjamas Wongsatayanon**

Thailand

**Lisa Wood**

USA

**Julia Woodman**

United Kingdom

**Liming Wu**

China

**Chung-Hsuen Wu**

Taiwan

**Ming-Jiuan Wu**

Taiwan

**Ching-Shuang Wu**

Taiwan

**Ming Wu**

USA

**Tie Wu**

China

**Leder Xavier**

Brazil

**Chunmei Xia**

China

**Daozong Xia**

China

**Jing Xianghong**

China

**Xiao He Xiao**

China

**Cheng Xiao**

China

**Xiaohe Xiao**

China

**Yin Xiao**

Australia

**Linshen Xie**

China

**Wei-Fen Xie**

China

**Ye Xing-Ming**

China

**Xingjiang Xiong**

China

**Guangwu Xiong**

China

**Xin-Gui Xiong**

China

**Zhang Xm**

China

**Xudong Xu**

China

**Lin Xu**

Hong Kong

**Zhaohui Xu**

China

**Qianqian Xu**

China

**Shi-Wen Xu**

China

**Bin Xu**

China

**Nik Soriani Yaacob**

Malaysia

**Toshihiko Yada**

Japan

**Arun Yadav**

India

**Toru Yago**

Japan

**Badrul Yahaya**

Malaysia

**Sien-Hung Yang**

Taiwan

**Guo-Yuan Yang**

China

**Wen-Chin Yang**

Taiwan

**Woong Mo Yang**

South Korea

**Cai-Guang Yang**

China

**Guangxiao Yang**

China

**Ling Yang**

China

**Zhang Yanjun**

China

**Wei Boon Yap**

Malaysia

**Jwu-Lai Yeh**

Taiwan

**Feng-Lin Yen**

Taiwan

**Hung-Rong Yen**

Taiwan

**Louis Yen**

USA

**Rakiswendé Serge Yerbanga**

Burkina Faso

**Beow Chin Yiap**

Malaysia

**Liusong Yin**

USA

**Yanyan Yin**

China

**Masahiro Yoneda**

Japan

**Audrey Yong**

Malaysia

**Seong Woo Yoon**

South Korea

**Takemi Yoshida**

Japan

**Zongbing You**

USA

**Junsang Yu**

South Korea

**Wen-Jen Yu**

Taiwan

**Shu Juan Yu**

China

**Jianbo Yu**

China

**Ying Yu**

China

**Zhi-Ling Yu**

Hong Kong

**Shuanghu Yuan**

China

**Yuan Yuan**

China

**Jong Won Yun**

South Korea

**Andrey Yurkov**

Germany

**Yasim Yusof**

Malaysia

**Zhonglin Yang**

China

**Fatheya Zahran**

Saudi Arabia

**Bojan Zalar**

Slovenia

**Alireza Zamani**

Iran

**Aleksander Zampronio**

Brazil

**Nelo Zanchi**

Brazil

**Antonia Zapantis**

USA

**Zied Zarai**

Tunisia

**Xiao Zhai**

China

**Yan Zhang**

China

**Yongyu Zhang**

China

**Min Zhang**

USA

**Ruixin Zhang**

USA

**Wei-Dong Zhang**

China

**Xuemei Zhang**

China

**Yan Zhang**

USA

**Ling Zhang**

USA

**Lei Zhang**

United Kingdom

**Li Zhang**

China

**Wei Zhang**

China

**Yonghui Zhang**

China

**Shijun Zhang**

China

**Naiqian Zhao**

China

**Kaicun Zhao**

United Kingdom

**Ying-Yong Zhao**

USA

**Guo-Qing Zheng**

China

**Guoping Zheng**

Australia

**Yiwen Zheng**

New Zealand

**Mingshan Zheng**

China

**Sihao Zheng**

China

**Linda Zhong**

Hong Kong

**Sen Zhong**

China

**Shanshan Zhou**

China

**Chunyan Zhou**

China

**Guang-Zhou Zhou**

China

**Hong-Hao Zhou**

China

**Kequan Zhou**

USA

**Wei Zhou**

USA

**Lei Zhou**

China

**Zhiqin Zhou**

China

**Bing Zhu**

China

**Liancai Zhu**

China

**Muhammad Zia-Ul-Haq**

Pakistan

**Suzanna Zick**

USA

**Malek Zihlif**

Jordan

**Lada Zivkovic**

Serbia

**Denis Zofou**

Cameroon

**Stefan Zorad**

Slovakia

**Yuxiao Zou**

China

**Ping Zou**

Japan

**Yuan Zou**

China

**Tycho Zuzak**

Germany

